# Supplementary material for: Multispecies-coadsorption-induced rapid preparation of graphene glass fiber fabric and applications in flexible pressure sensor
Source: Nat Commun. 2024 Jun 12;15:5040. doi: 10.1038/s41467-024-48958-y (PMC11169262; doi:10.1038/s41467-024-48958-y)
Supplement: Supplementary file 1 — Supplementary information [file 41467_2024_48958_MOESM1_ESM.pdf]

Supplementary Information for:

Multispecies-Coadsorption-Induced Rapid Preparation of Graphene Glass Fiber Fabric and Applications in Flexible Pressure Sensor

Kun Wang[1,7], Xiucai Sun[1,2,7], Shuting Cheng[2,3,6], Yi Cheng[1], Kewen Huang[1], Ruojuan Liu[1,2], Hao Yuan[1,2], Wenjuan Li[1,2], Fushun Liang[1,2], Yuyao Yang[1,2], Fan Yang[1,2], Kangyi Zheng[2,4], Zhiwei Liang[2,5], Ce Tu[2], Mengxiong Liu[1,2], Mingyang Ma[1,2], Yunsong Ge[1,2], Muqiang Jian[1,2,6], Wanjian Yin[2,4], Yue Qi[2]*, Zhongfan Liu[1,2]*

[1] Centre for Nanochemistry, Beijing Science and Engineering Centre for Nanocarbons, Beijing National Laboratory for Molecular Sciences, College of Chemistry and Molecular Engineering, Peking University, Beijing 100871, China.

[2] Beijing Graphene Institute (BGI), Beijing 100095, China.

[3] State Key Laboratory of Heavy Oil Processing, College of Science, China University of Petroleum, Beijing 102249, China.

[4] College of Energy, Soochow Institute for Energy and Materials Innovations (SIEMIS), Jiangsu Provincial Key Laboratory for Advanced Carbon Materials and Wearable Energy Technologies, Soochow University, Suzhou 215006, China.

[5] Guangdong Provincial Key Laboratory of Quantum Engineering and Quantum Materials, School of Physics, South China Normal University, Guangzhou 510006, China

[6] Key Laboratory of Organic Optoelectronics and Molecular Engineering of the Ministry of Education, Department of Chemistry, Tsinghua University, Beijing 100084, China.

[7] These authors contributed equally: Kun Wang, Xiucai Sun, Shuting Cheng.

* Correspondence: zfliu@pku.edu.cn; qiyue@bgi-graphene.com

This PDF file includes:

Supplementary Figs. 1−20

Supplementary Table 1 and 2

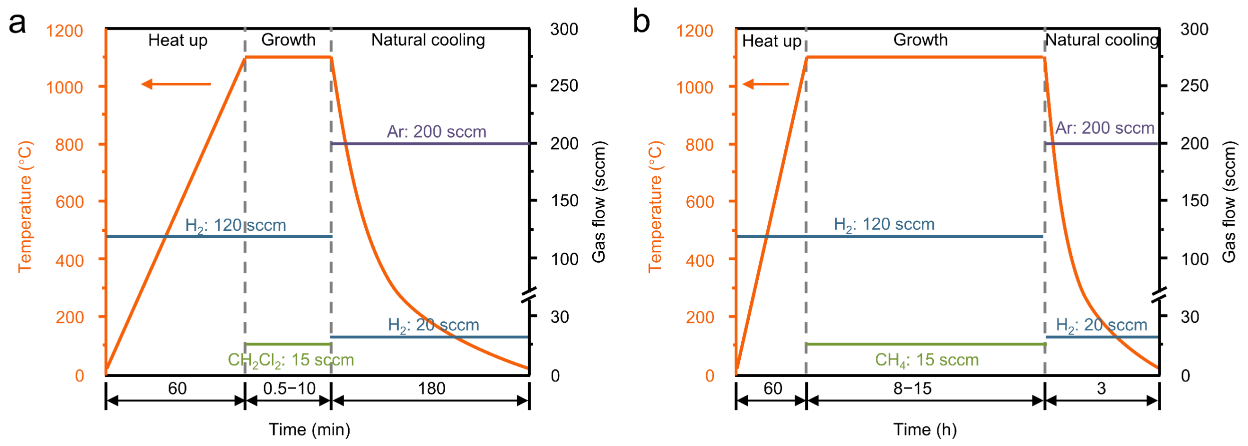

Supplementary Fig. 1 | Typical chemical vapor deposition (CVD) graphene growth process in (**a**) dichloromethane and (**b**) methane CVD growth system. In a typical procedure, the system was evacuated to a base pressure of <1 Pa and heated to ~1100 °C under a H_2 flow of 120 sccm. Subsequently, dichloromethane vapor or methane with desired flow (5−24 sccm) was pumped to the chamber for the graphene growth. Throughout the growth process, the chamber pressure was held at approximately 400−500 Pa depending on the flow of dichloromethane vapor or methane. The growth of graphene lasted for 0.5−10 min for dichloromethane and 8−15 h for methane, followed by natural cooling process to room temperature under a H_2 flow of 20 sccm and Ar flow of 200 sccm.

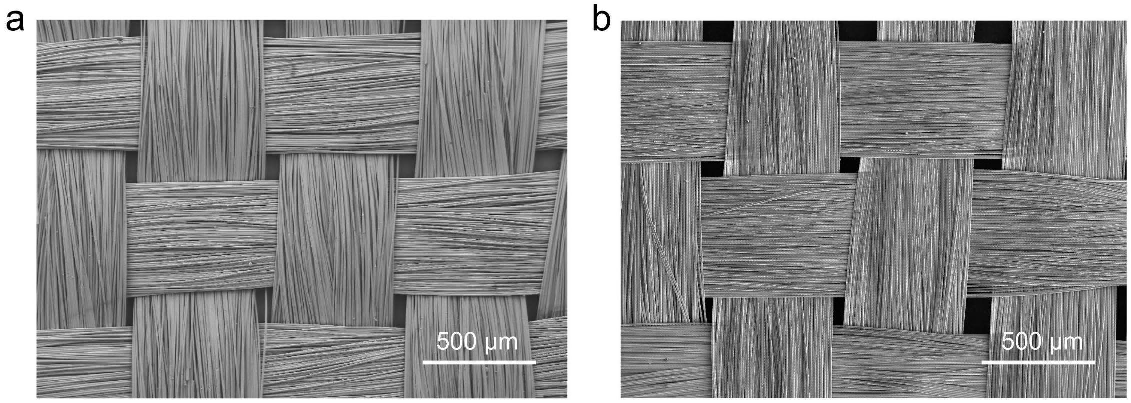

Supplementary Fig. 2 | Scanning electron microscopy (SEM) images of (**a**) original glass fiber fabric (GFF) and (**b**) graphene glass fiber fabric (GGFF) obtained after the high-temperature (~1100 °C) graphene chemical vapor deposition (CVD) growing process. The structure of the fiber was well maintained after the high-temperature treatment.

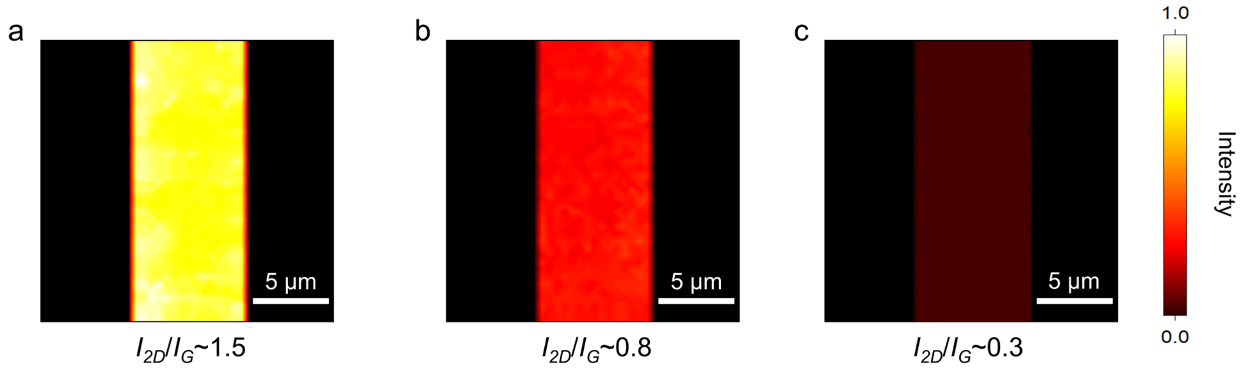

Supplementary Fig. 3 | Intensity of Raman *2D* peak mappings of the fabricated graphene glass fiber fabric (GGFF) with graphene thickness of (**a**) ~2 nm, (**b**) ~15 nm, and (**c**) ~45 nm. The *2D* peak intensity of graphene ribbons in (b, c) was normalized by that of graphene ribbon in (a).

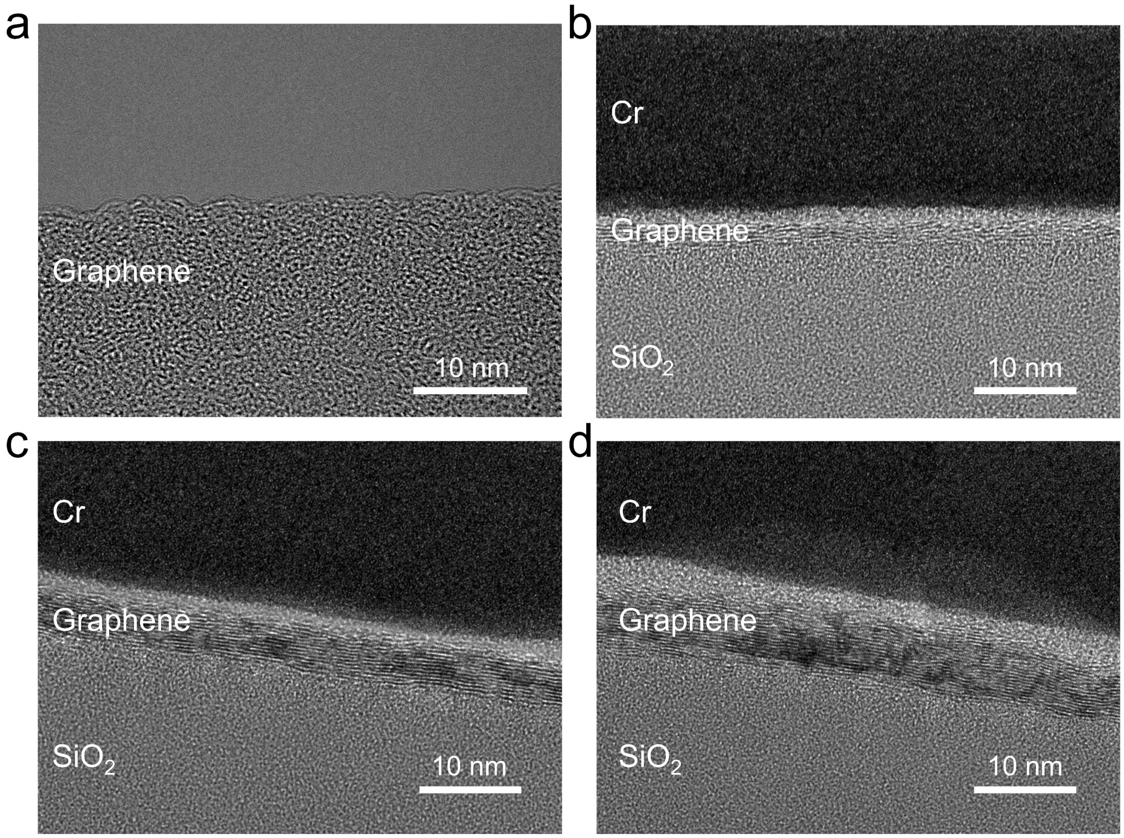

Supplementary Fig. 4 | Transmission electron microscope (TEM) images of graphene grown on glass fiber with different layer thickness. Growth parameters: growth temperature of ~1100 °C, H_2 flow of 120 sccm, CH_2Cl_2 flow of 15 sccm, growth time of (**a**) ~30 s, (**b**) 40 s, (**c**) 1 min, and (**d**) 2 min. (a) was the TEM image of monolayer graphene transferred onto a TEM grid. (b−d) Cross-sectional TEM images of graphene glass fiber fabric (GGFF) samples prepared by focused ion beam (FIB) techniques with chromium (Cr) layers deposited to protect samples from possible damage during sample preparation.

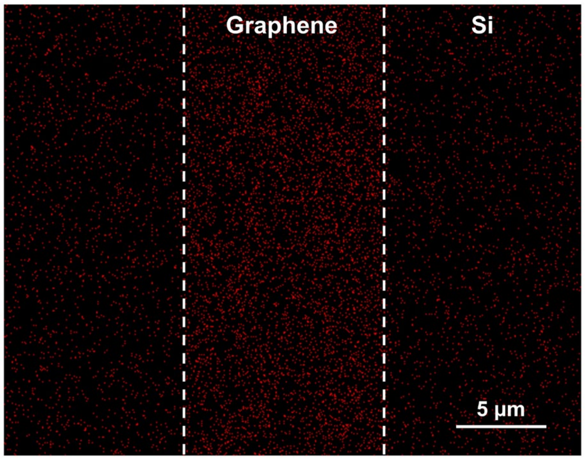

Supplementary Fig. 5 | Energy-dispersive X-ray (EDX) mapping of C element for the corresponding graphene ribbon in Fig. 1d (transferred onto the silicon substrate).

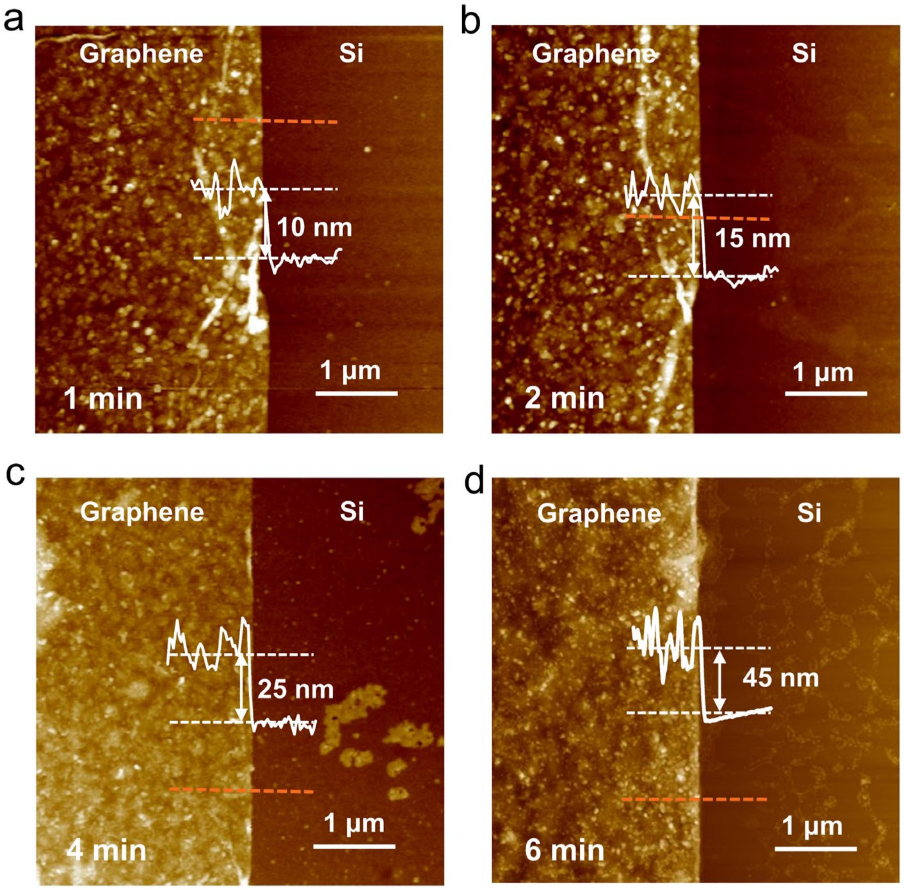

Supplementary Fig. 6 | Atomic force microscopy (AFM) characterizations of graphene ribbons (measured after being transferred onto the silicon substrate) with different thicknesses obtained through growth time modulation during graphene chemical vapor deposition (CVD) growth process.

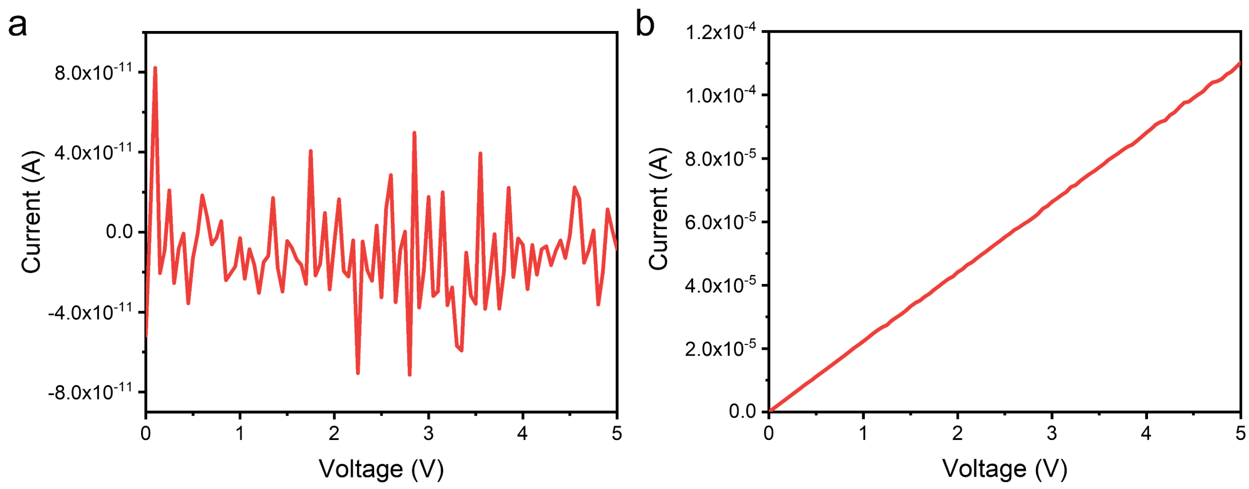

Supplementary Fig. 7 | Current-voltage curves of graphene glass fiber fabrics (GGFFs) with growth time of (**a**) 15 s and (**b**) 30 s, showing open-circuit (corresponding to period I in Fig. 2) and connected-circuit (corresponding to period II in Fig. 2) characteristics, respectively.

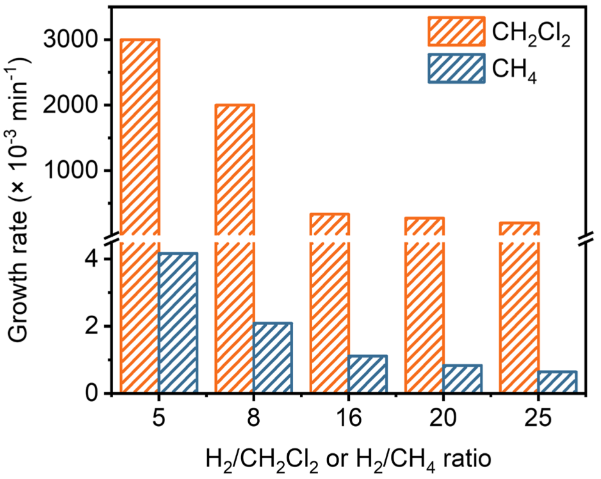

Supplementary Fig. 8 | Growth rates of graphene grown with dichloromethane and methane at the different H_2/CH_2Cl_2 or H_2/CH_4 ratios. The growth rates of graphene on glass fiber fabric (GFF) in the dichloromethane chemical vapor deposition (CVD) system kept at ~3 orders of magnitude higher that of methane.

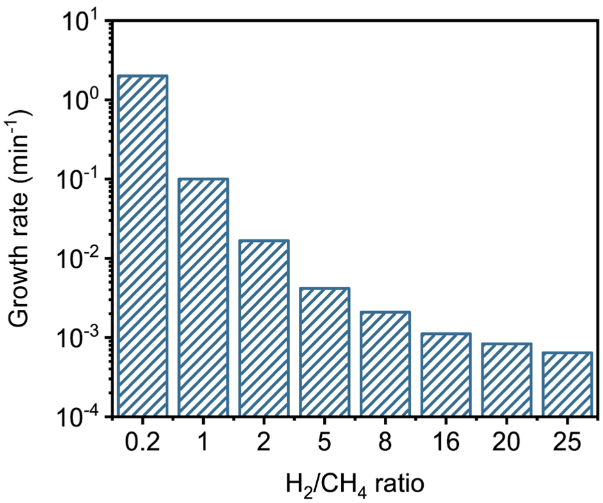

Supplementary Fig. 9 | Growth rates of methane-grown graphene at different H_2/CH_4 ratios, revealing the increased growth rate with H_2/CH_4 ratio reducing.

In graphene chemical vapor deposition (CVD) growth system with methane as the carbon precursor, the graphene growth rate can be effectively manipulated by adjusting the H_2/CH_4. For example, as shown in Supplementary Fig. 9, when H_2/CH_4 ratio was modulated from 0.2 to 25, the graphene growth rate can be manipulated from ~6.4×10^{-4} to 2 min^{-1}. Specifically, the growth rate of dichloromethane-grown graphene in Fig. 2d was ~2 min^{-1} (prepared under H_2/CH_2Cl_2 ratio of 8:1, with H_2 flow of 120 sccm and CH_2Cl_2 vapor flow of 15 sccm, CVD growth time ~0.5 min). To get the same growth rate as that of dichloromethane-grown graphene, H_2/CH_4 ratio used for methane-grown graphene in Fig. 2e was set at 1:5 (with methane flow of 50 sccm and H_2 flow of 10 sccm, CVD growth time ~0.5 min), and from the data in Supplementary Fig. 9, at this H_2/CH_4 ratio, the graphene growth rate was also ~2 min^{-1}.

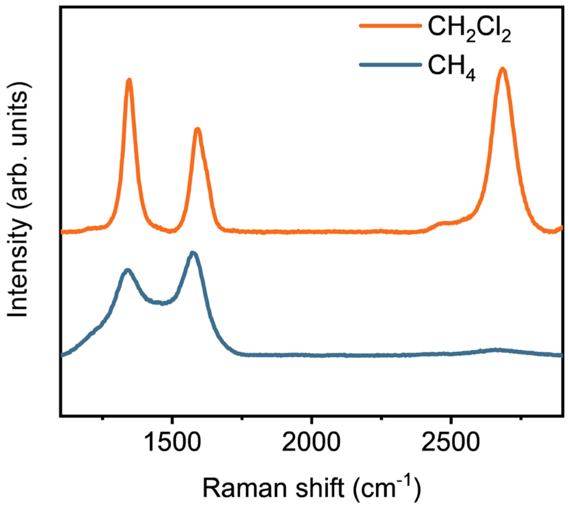

Supplementary Fig. 10 | Comparison of Raman spectra for dichloromethane- and methane-grown graphene obtained with the same growth rate. In contrast to the characteristic Raman *2D* band (~2,680 cm^{-1}) for dichloromethane-grown graphene, the Raman *2D* band of methane-grown graphene is nearly negligible.

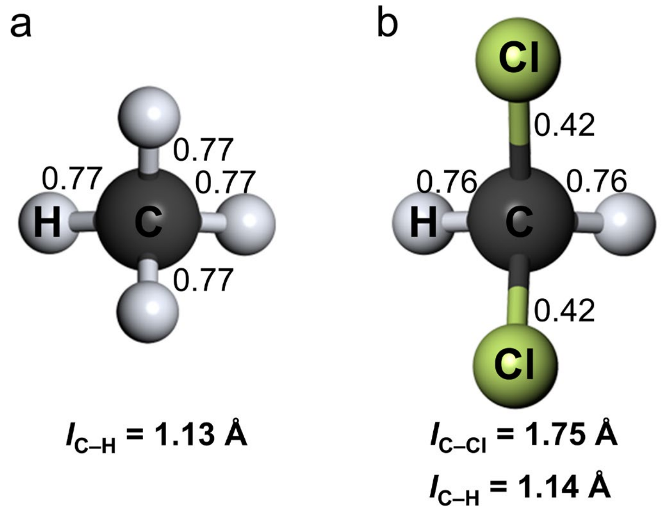

Supplementary Fig. 11 | Density-functional theory (DFT) calculated bond lengths and overlap populations of (**a**) C−H in methane molecule and (**b**) C−H and C−Cl in dichloromethane molecule. The overlap population between two atoms provides a measure of the extent of electron density overlap between their respective atomic orbitals, indicating the strength of the bond between those atoms.

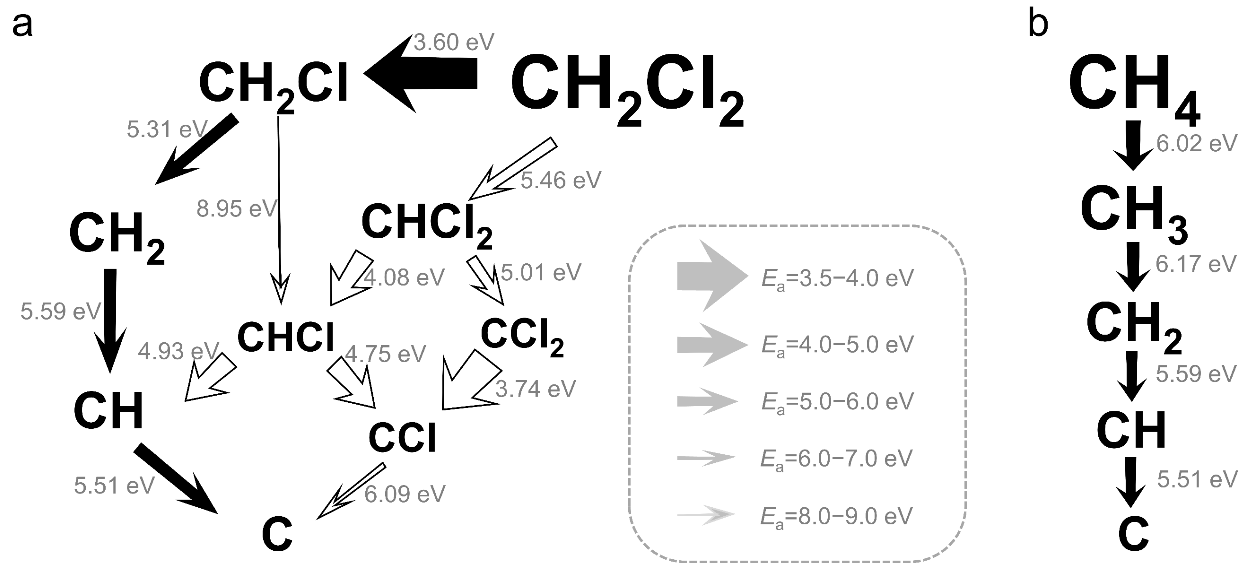

Supplementary Fig. 12 | Kinetic calculations of dichloromethane and methane pyrolysis. a, Energy barriers E_a (in eV) and possible routes for dichloromethane pyrolysis in gas phase. The black filled arrows indicate the main decomposition route. **b,** Energy barrier of CH_4 dehydrogenation processes in gas phase.

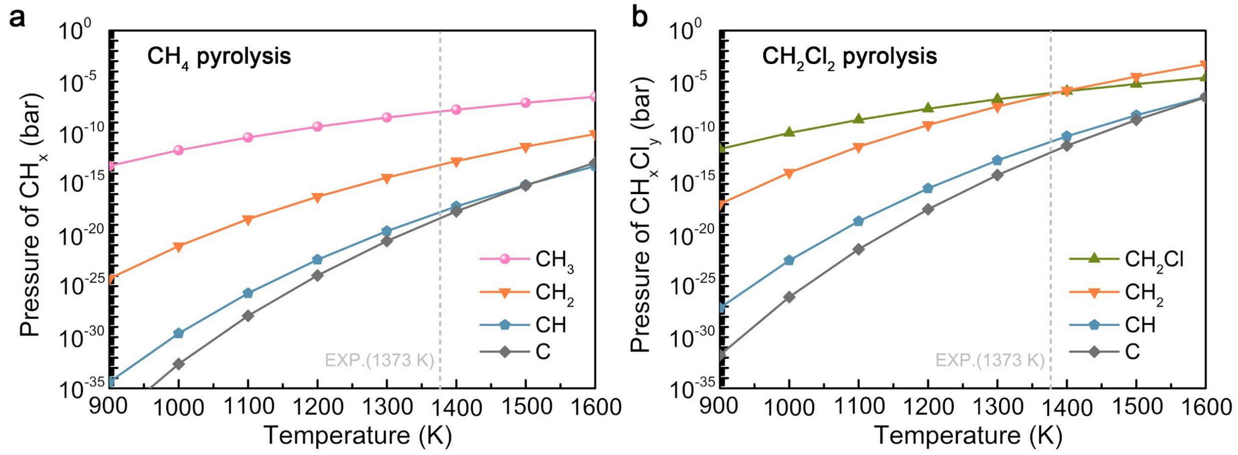

Supplementary Fig. 13 | Calculated partial pressure of the decomposition products of (**a**) methane and (**b**) dichloromethane as a function of temperature. The reaction is assumed under a thermodynamic equilibrium, and $P(CH_4) = P(CH_2Cl_2) = 2.5 \times 10^{-4}$ bar, $P(H_2) = 2.0 \times 10^{-3}$ bar. The typical graphene growth temperature of ~1100 °C (1373 K) is marked with grey line.

At the experimental temperature of 1373 K, the partial pressure of CH_3, the main product of CH_4 pyrolysis, was calculated to be 1.82×10^{-8} bar, whereas the partial pressure of CH_2, the dominant active species in CH_2Cl_2 system, was calculated to be 1.37×10^{-6} bar. Therefore, CH_2Cl_2 was 10^2 times more capable than CH_4 of providing the active carbon species for the same fluxes of carbon source.

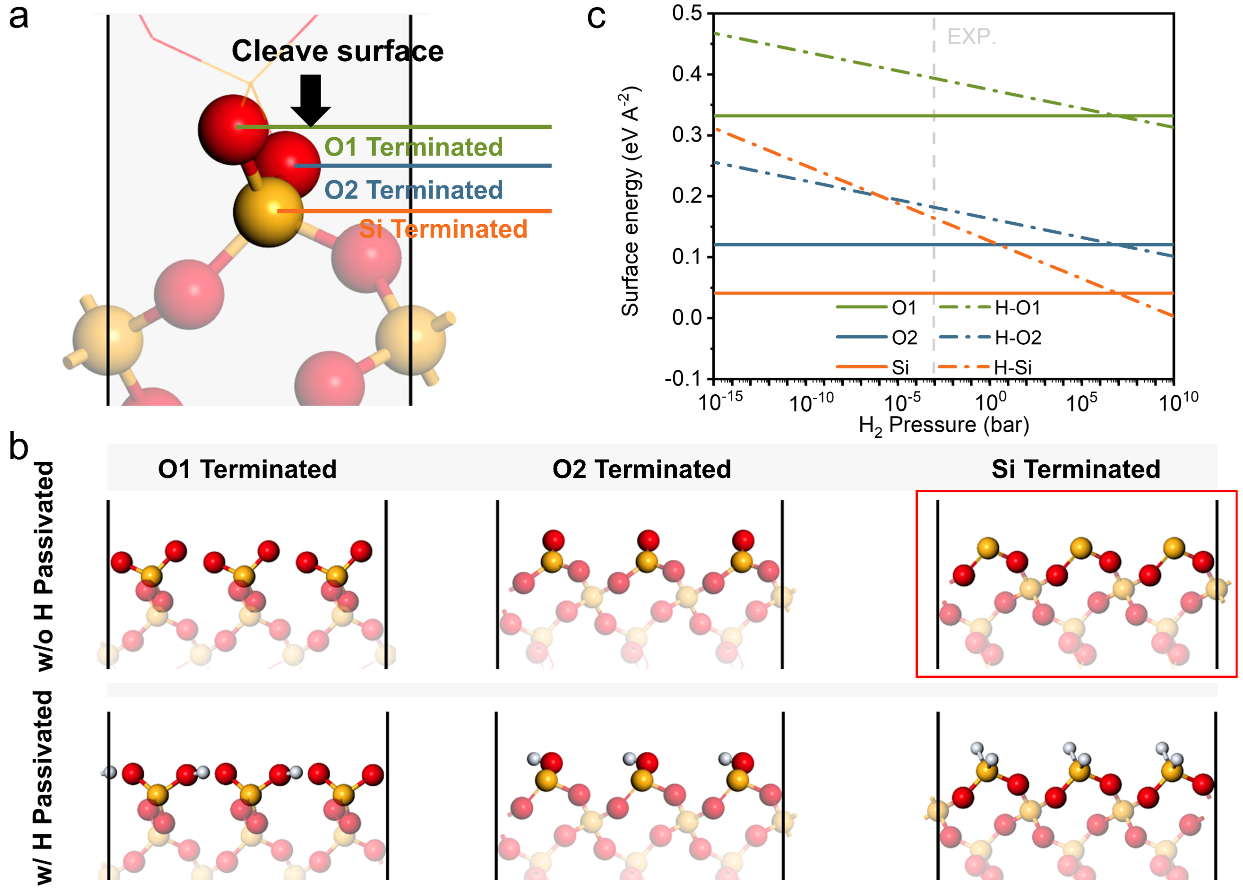

Supplementary Fig. 14 | Calculation model of the glass fiber fabric (GFF) substrate. a, Schematic of the atomic configurations for $SiO_2(0001)$ surfaces terminated with different elements. **b,** Stable configurations of O1-, O2-, and Si-terminated surface structures with or without H passivation. **c,** Comparisons of the surface energy corresponding to the six configurations in (b), where Si-terminated $SiO_2(0001)$ without H passivation (marked by the red box in (b)) is proved to be the most stable structure with the lowest surface energy.

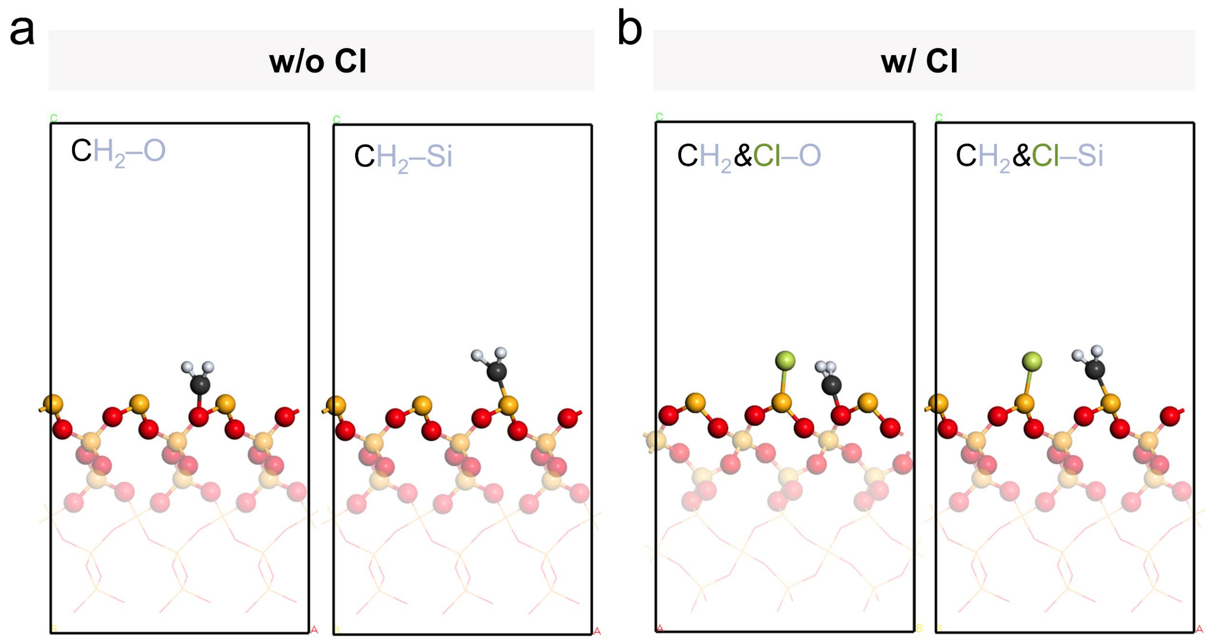

Supplementary Fig. 15 | Stable configurations and adsorpstion energies (E_{ads}) of CH_2 adsorbed on $SiO_2(0001)$ surface (**a**) without and (**b**) with Cl coadsorption.

Supplementary Table 1 | Comparison of adsorption energies (E_{ads}) on the SiO_2 surface for the dominant carbon species (CH_3, formed by methane pyrolysis, and CH_2 produced by dichloromethane pyrolysis). The thermodynamic stability of the adsorption increases with decreasing negative E_{ads}. Without Cl coadsorption, the adsorption energy of CH_2 is ~−1.24 eV for Si site and ~1.40 eV for O site, revealing the nonactivity of O site for CH_2 adsorption. With Cl coadsorption on Si site, the adsorption energy of CH_2 decreases from ~−1.24 eV to ~−1.26 eV for Si site and from ~1.40 eV to ~−0.63 eV for adjacent non-bonding O site. The significant decrease of adsorpstion energies on O site indicates that CH_2 adsorption is greatly enhanced and the nonactive O site is thus activated.

	**CH_4**	**CH_2Cl_2**
Dominant active carbon species	CH_3	CH_2
E_{ads}(eV)	−0.60 (CH_3-Si)	1.40 (CH_2−O)<br>−1.24 (CH_2−Si)<br>−0.63 (CH_2&Cl−O)<br>−1.26 (CH_2&Cl−Si)

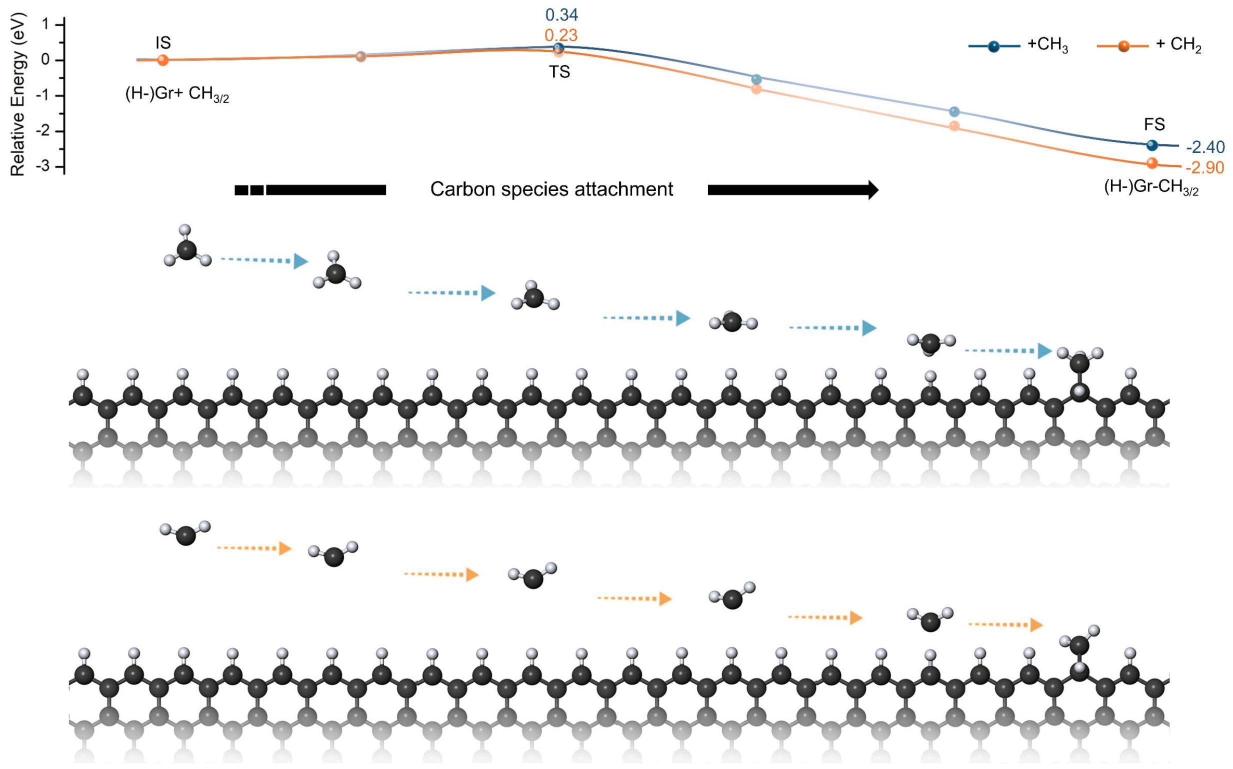

Supplementary Fig. 16 | Energy profiles and the corresponding structure evolution for CH_3 or CH_2 carbon species attachment at the growing zigzag graphene edge.

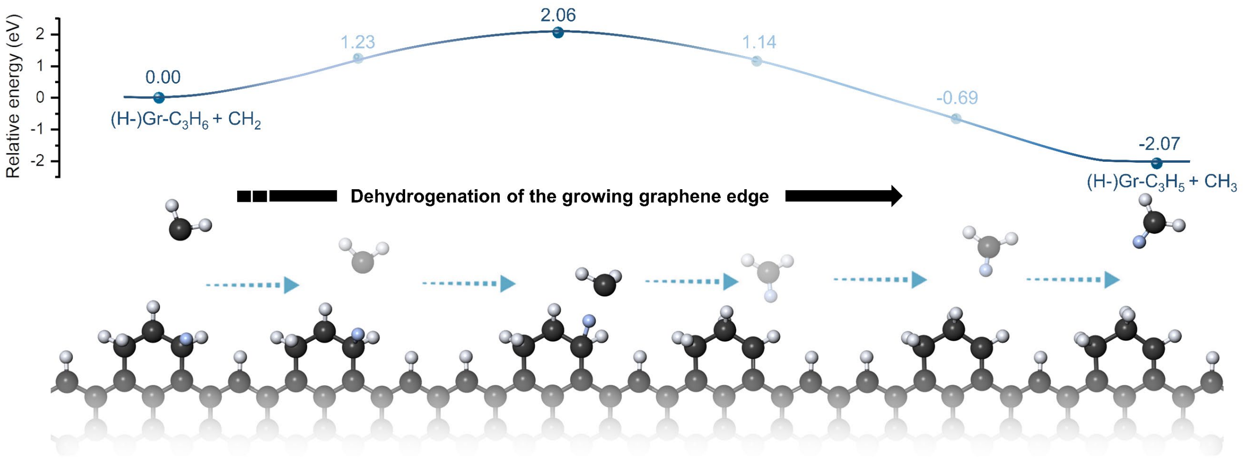

Supplementary Fig. 17 | Energy profiles and the corresponding structure evolution during the dehydrogenation kinetics of the growing zigzag edges of graphene domain, where (H-)Gr represents that the frontier edge of graphene is terminated by H. The H atoms at graphene edges are removed by CH_2 species in the gas phase via CH_2 + H−Gr → CH_3 + Gr reaction. The high reaction energy barrier of ~2.06 eV severely limits the expansion of graphene edges.

Supplementary Table 2 | Thermal and kinetic parameters of two carbon sources involved in graphene growth.

		CH_4	CH_2Cl_2
Dominant active carbon species		CH_3	CH_2
Carbon species attachment	ΔE (eV)	2.40	−2.90
	E_a (eV)	−0.34	0.23
Growing edge dehydrogenation	ΔE (eV)	−2.07	−0.63
	E_a (eV)	2.06	0

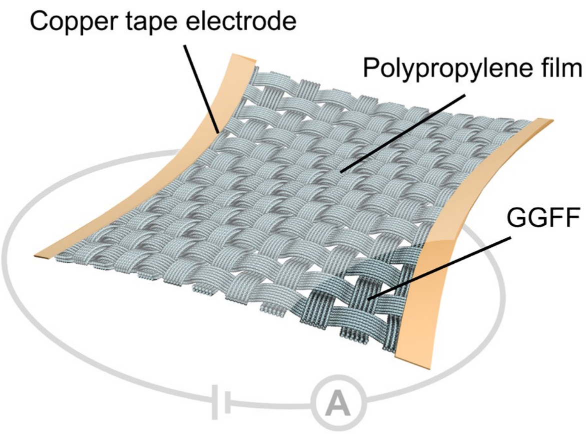

Supplementary Fig. 18 | Schematic of the graphene glass fiber fabric (GGFF) flexible pressure sensor, where GGFF is encapsulated by polypropylene films.

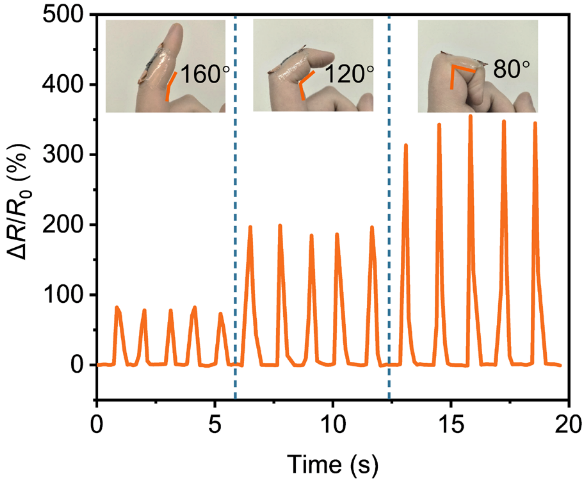

Supplementary Fig. 19 | $\Delta R/R_0$ of graphene glass fiber fabric (GGFF) pressure sensor at different finger bending angles, exhibiting rapid variations of relative resistance with good repeatability and stability.

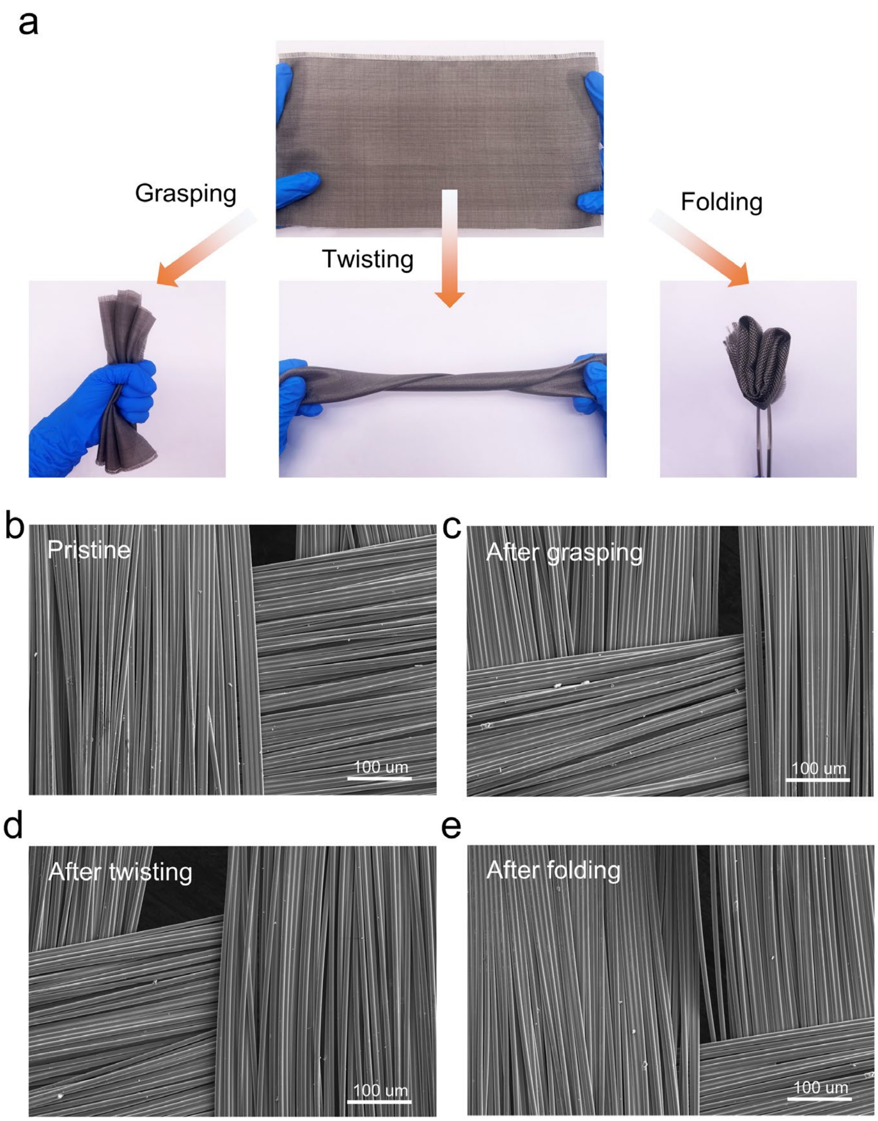

Supplementary Fig. 20 | a Photographs of graphene glass fiber fabric (GGFF) being twisted, grasped, and folded; **b−e** Scanning electron microscopy (SEM) images of (b) pristine GGFF and GGFF after being (c) grasped, (d) twisted, and (e) folded (every deformation action was repeated for 100 times). After the repeated mechanical deformations, the morphology of GGFF presented negligible change and no peeling of graphene layers was observed.
