# Supplementary material for: Multispecies-coadsorption-induced rapid preparation of graphene glass fiber fabric and applications in flexible pressure sensor
Source: Nat Commun. 2024 Jun 12;15:5040. doi: 10.1038/s41467-024-48958-y (PMC11169262; doi:10.1038/s41467-024-48958-y)
Supplement: Supplementary file 3 — Reporting Summary [file 41467_2024_48958_MOESM3_ESM.pdf]

nature portfolio

Corresponding author(s): Yue Qi, Zhongfan Liu

Last updated by author(s): Apr 17, 2024

Reporting Summary

Nature Portfolio wishes to improve the reproducibility of the work that we publish. This form provides structure for consistency and transparency in reporting. For further information on Nature Portfolio policies, see our Editorial Policies and the Editorial Policy Checklist.

Statistics

For all statistical analyses, confirm that the following items are present in the figure legend, table legend, main text, or Methods section.

n/a	Confirmed	
☐	☒	The exact sample size (n) for each experimental group/condition, given as a discrete number and unit of measurement
☐	☒	A statement on whether measurements were taken from distinct samples or whether the same sample was measured repeatedly
☐	☒	The statistical test(s) used AND whether they are one- or two-sided<br>*Only common tests should be described solely by name; describe more complex techniques in the Methods section.*
☒	☐	A description of all covariates tested
☒	☐	A description of any assumptions or corrections, such as tests of normality and adjustment for multiple comparisons
☐	☒	A full description of the statistical parameters including central tendency (e.g. means) or other basic estimates (e.g. regression coefficient) AND variation (e.g. standard deviation) or associated estimates of uncertainty (e.g. confidence intervals)
☒	☐	For null hypothesis testing, the test statistic (e.g. F, t, r) with confidence intervals, effect sizes, degrees of freedom and P value noted<br>*Give P values as exact values whenever suitable.*
☒	☐	For Bayesian analysis, information on the choice of priors and Markov chain Monte Carlo settings
☒	☐	For hierarchical and complex designs, identification of the appropriate level for tests and full reporting of outcomes
☒	☐	Estimates of effect sizes (e.g. Cohen's d, Pearson's r), indicating how they were calculated

Our web collection on statistics for biologists contains articles on many of the points above.

Software and code

Policy information about availability of computer code

Data collection	Vienna Ab initio Simulation Package (VASP) software
Data analysis	VASP software was used to conduct density function theory (DFT) calculations about the molecular characteristics of dichloromethane and its participation in the elementary processes of graphene CVD growth.

For manuscripts utilizing custom algorithms or software that are central to the research but not yet described in published literature, software must be made available to editors and reviewers. We strongly encourage code deposition in a community repository (e.g. GitHub). See the Nature Portfolio guidelines for submitting code & software for further information.

Data

Policy information about availability of data

All manuscripts must include a data availability statement. This statement should provide the following information, where applicable:

- Accession codes, unique identifiers, or web links for publicly available datasets
- A description of any restrictions on data availability
- For clinical datasets or third party data, please ensure that the statement adheres to our policy

Provide your data availability statement here.

Research involving human participants, their data, or biological material

Policy information about studies with human participants or human data. See also policy information about sex, gender (identity/presentation), and sexual orientation and race, ethnicity and racism.

Reporting on sex and gender	Two volunteers participated in the GGFF pressure sensor experiments, comprising a 27-year-old male for finger bending and pulse signal detections and a 26-year-old female for human vocal signal detection. The gender of participants was determined based on self-report. The gender was not considered in the study design because the sensitivity of pressure sensors has no certain relationship with gender. The data were obtained with the informed consent of all participants,
Reporting on race, ethnicity, or other socially relevant groupings	In our study, we do not consider race, ethnicity, or other socially relevant groupings as variables, which have no certain relationship with the sensitivity of pressure sensors.
Population characteristics	There is no certain relationship between the sensitivity of pressure sensors and population characteristics.
Recruitment	The participants volunteer willingly, and their involvement does not bias the results.
Ethics oversight	The data were obtained with the informed consent of all participants. All human experiments were performed in compliance with the protocol approved by the Institutional Review Board of Tsinghua University.

Note that full information on the approval of the study protocol must also be provided in the manuscript.

Field-specific reporting

Please select the one below that is the best fit for your research. If you are not sure, read the appropriate sections before making your selection.

☐ Life sciences ☐ Behavioural & social sciences ☒ Ecological, evolutionary & environmental sciences

For a reference copy of the document with all sections, see nature.com/documents/nr-reporting-summary-flat.pdf

Ecological, evolutionary & environmental sciences study design

All studies must disclose on these points even when the disclosure is negative.

Study description	We designed a novel strategy for large-scale, rapid chemical vapor deposition (CVD) growth of graphene on noncatalytic insulating glass fiber fabric. The use of dichloromethane as a carbon precursor in the CVD system enabled precise modulation of multi-elementary processes during graphene growth, which achieved a substantial increase in growth rate and carbon utilization compared to conventional methane precursors. The resulting graphene glass fiber fabric exhibited excellent electrical conduction and a hierarchical conductive configuration, making it a promising candidate for lightweight, flexible sensors with high sensitivity and portability in human motion and physiological signal monitoring.
Research sample	The commercially available glass fiber fabric was purchased from Wuhan Sino Type Optoelectronic Technology CO., LTD. The details of research samples are listed in Method section.
Sampling strategy	No sample sizes were calculated. The graphene glass fiber fabric was prepared through the direct CVD graphene growth on glass fiber fabric substrates.
Data collection	Kun Wang, Shuting Cheng, Yi Cheng, Kewen Huang, Hao Yuan, Wenjuan Li, Yuyao Yang, Fan Yang, Kangyi Zheng, Zhiwei Liang, Mengxiong Liu, Mingyang Ma, Yunsong Ge, Muqiang Jian and Wanjian Yin performed the SEM, TEM, XPS, Raman and sheet resistance characterization. Xiucai Sun and Kun Wang studied the mechanism of rapid growth of graphene by dichloromethane CVD system. Kun Wang, Shuting Cheng, Ruojuan Liu, Fushun Liang and Ce Tu made the GGFF-based flexible pressure sensor and tested its sensitivity.
Timing and spatial scale	We first conceived the idea and conducted the experiment on Nov. 16th, 2021, and all experiments have been finished on Nov. 10th, 2023.
Data exclusions	No data excluded.
Reproducibility	All attempts for the reproducibility were successful.
Randomization	The allocation is random.
Blinding	The blinding is possible.

Did the study involve field work? ☐ Yes ☒ No

Reporting for specific materials, systems and methods

We require information from authors about some types of materials, experimental systems and methods used in many studies. Here, indicate whether each material, system or method listed is relevant to your study. If you are not sure if a list item applies to your research, read the appropriate section before selecting a response.

Materials & experimental systems

n/a	Involved in the study
☒	☐ Antibodies
☒	☐ Eukaryotic cell lines
☒	☐ Palaeontology and archaeology
☒	☐ Animals and other organisms
☒	☐ Clinical data
☒	☐ Dual use research of concern
☒	☐ Plants

Methods

n/a	Involved in the study
☒	☐ ChIP-seq
☒	☐ Flow cytometry
☒	☐ MRI-based neuroimaging

Plants

Seed stocks	*Report on the source of all seed stocks or other plant material used. If applicable, state the seed stock centre and catalogue number. If plant specimens were collected from the field, describe the collection location, date and sampling procedures.*
Novel plant genotypes	*Describe the methods by which all novel plant genotypes were produced. This includes those generated by transgenic approaches, gene editing, chemical/radiation-based mutagenesis and hybridization. For transgenic lines, describe the transformation method, the number of independent lines analyzed and the generation upon which experiments were performed. For gene-edited lines, describe the editor used, the endogenous sequence targeted for editing, the targeting guide RNA sequence (if applicable) and how the editor was applied.*
Authentication	*Describe any authentication procedures for each seed stock used or novel genotype generated. Describe any experiments used to assess the effect of a mutation and, where applicable, how potential secondary effects (e.g. second site T-DNA insertions, mosiacism, off-target gene editing) were examined.*
