# Supplementary material for: Multispecies-coadsorption-induced rapid preparation of graphene glass fiber fabric and applications in flexible pressure sensor
Source: Nat Commun. 2024 Jun 12;15:5040. doi: 10.1038/s41467-024-48958-y (PMC11169262; doi:10.1038/s41467-024-48958-y)
Supplement: Supplementary file 4 — Description of Additional Supplementary Files [file 41467_2024_48958_MOESM4_ESM.pdf]

Description of Additional Supplementary Files

File Name: Supplementary Movie 1

Description: Resistance variations of graphene glass fiber fabric sensor attached on a loudspeaker playing a burst of birdsong.
